# Salvage Mini-Open Achilles Tendon Repair Using the Percutaneous Achilles Repair System (PARS): A Technical Tip

**DOI:** 10.7759/cureus.102742

**Published:** 2026-01-31

**Authors:** Sarah Oyadomari, Andrew R Hsu, Naudereh Noori

**Affiliations:** 1 Orthopaedic Surgery, University of California Irvine Medical Center, Orange, USA

**Keywords:** achilles tendinopathy, achilles tendon injury, achilles tendon repair, mini-open repair, percutaneous achilles repair system

## Abstract

Minimally invasive surgical repair options for mid-substance Achilles tendon ruptures may pose some limitations in regard to complex tear patterns or poor overall tendon quality. We have encountered cases where percutaneously passed sutures with the help of a targeting device fail to adequately capture the proximal tendon stump.

In this technical report, we report a salvage technique for such cases that prevents the need for conversion to an extensile open approach, relying on smaller mini-open incisions.

## Introduction

Achilles tendon ruptures are a common athletic injury occurring in 2.1 per 100,000 person-years, with a growing incidence in active older individuals [1,2]. Minimally invasive surgical options have evolved to mitigate the complications from traditional open surgical repairs, which disrupt local biology and carry the risk of greater wound complications [3,4]. The Percutaneous Achilles Repair System (PARS, Arthrex Inc., Naples, FL, USA) is one example of a commonly used minimally invasive technique to repair mid-substance Achilles tendon ruptures [5]. However, if the proximal tendon stump has complex multiplanar tears or poor tendon quality, the repair sutures passed with the PARS jig may have insufficient purchase and pull through. In these situations, conversion to an extensile open approach is often performed to visualize and capture a greater extent of the damaged tendon; however, the authors have successfully devised an alternative, less invasive mini-open salvage technique detailed here. 

## Technical report

The patient is positioned prone on the operating table. A thigh tourniquet is used, and an approximately 2 cm incision is planned at the palpable defect. A longitudinal or transverse incision can be used. The PARS jig is used as described [5] to pass the repair sutures, which, when cycled, may pull through the tendon. This may be due to improper positioning of the jig, so it is advised to try multiple attempts. With persistent pull-through of the sutures, poor proximal tendon quality or complex tearing is likely the cause. 

**Figure 1:**
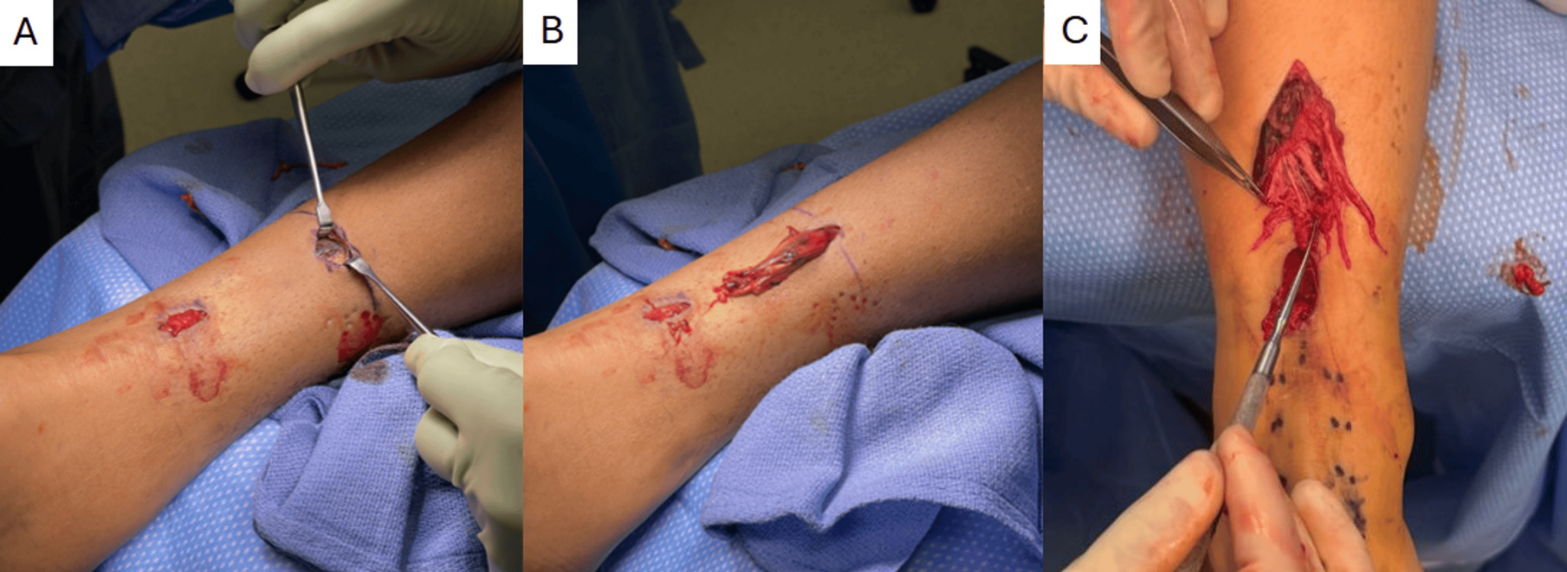
Counter incision (proximal) with intact paratenon exposed (A). Proximal tendon stump tunneled through showing evidence of delamination (B) and extensive fraying with longitudinal tears (C).

A mini-open 2-3 cm longitudinal counter incision is made proximal to the initial incision, leaving at least a 3 cm skin bridge (Figure 1). Of note, the summation of the counter incision and initial incision is smaller than the extensile extension needed to allow access to the same area of the proximal tendon. In some instances, the paratenon is ruptured at this region; in cases where it is not, an incision is made through the paratenon to access the tendon. The proximal tendon stump is then tunneled retrograde from the distal incision out of the proximal one (Figure 1). Figure 1 shows two examples of poor tendon quality with a coronal delamination and fraying with multiple longitudinal splits, both of which can account for the inability of the PARS jig to capture the tendon fully.

**Figure 2:**
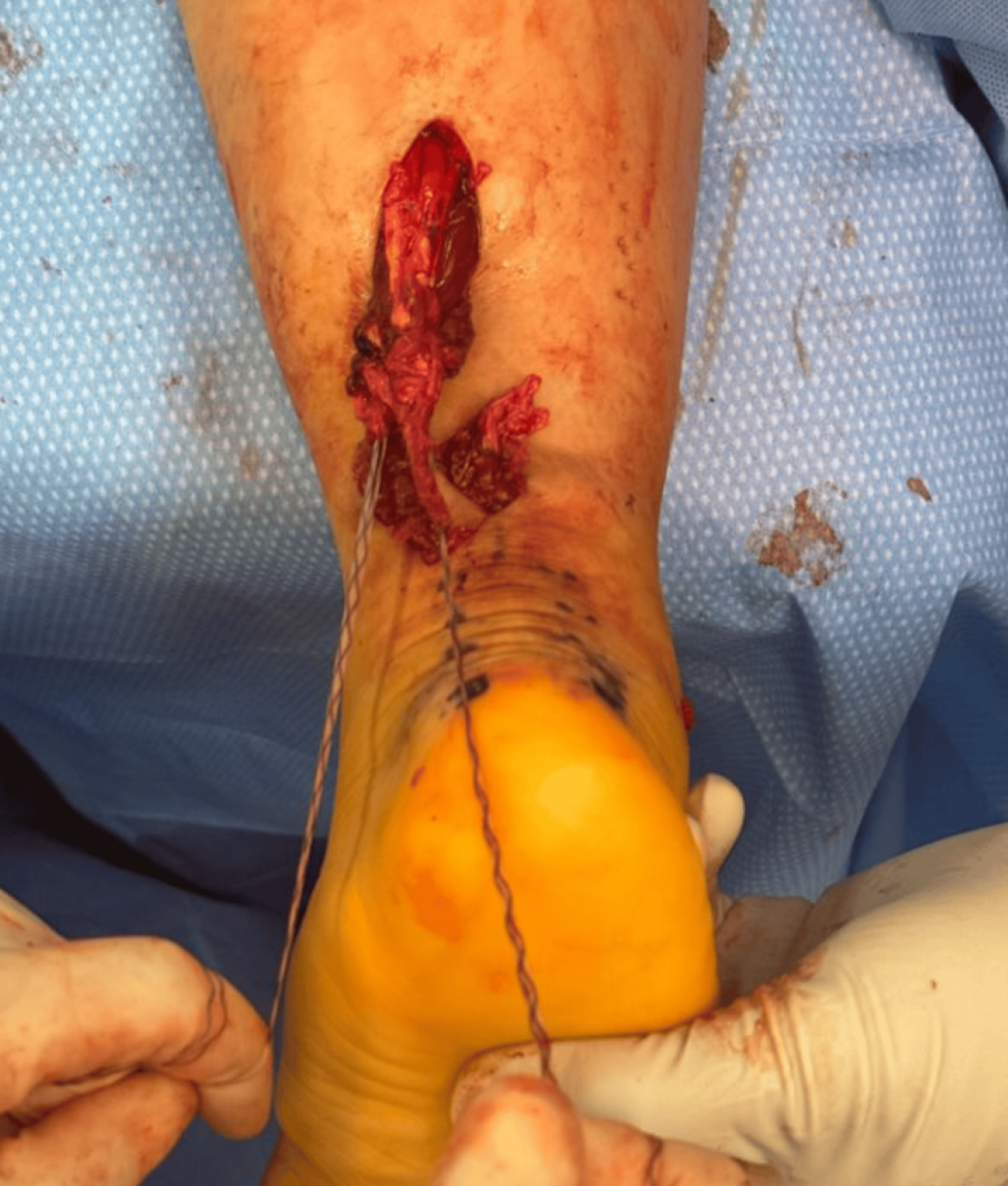
Four-strand repair with suture tape using a core weave pattern with good purchase.

A four-strand repair is completed with 1.3 mm SutureTape (Arthrex Inc., Naples, FL, USA) using a core weave (Figure 2) or Krackow repair technique. All sutures are cycled 10 times to remove creep and ensure tendon purchase. The suture ends and the proximal tendon stump are tunneled anterograde through the distal incision, preserving any remaining paratenon.

**Figure 3:**
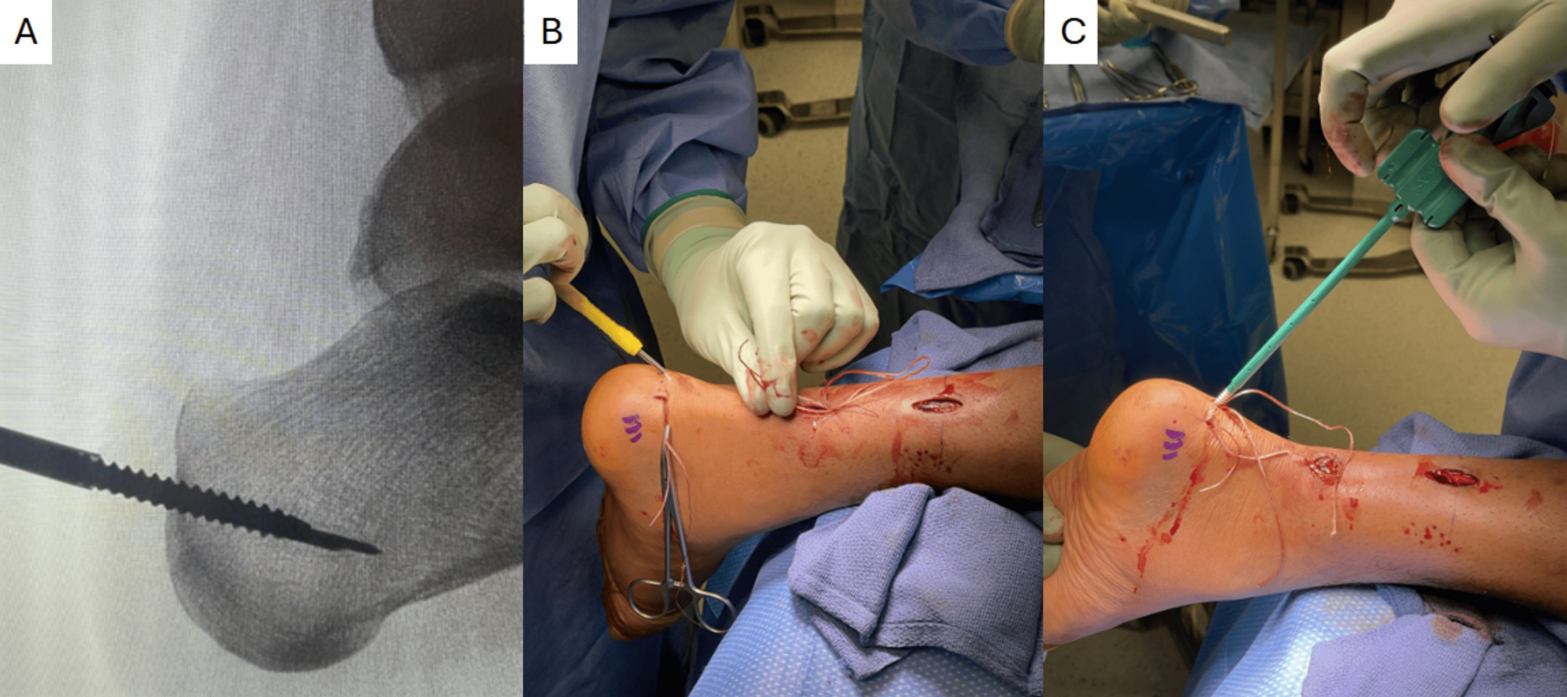
Appropriate positioning of calcaneal anchors confirmed on fluoroscopy (A), followed by tunneling through the distal stump (B) and insertion of anchors (C).

**Figure 4:**
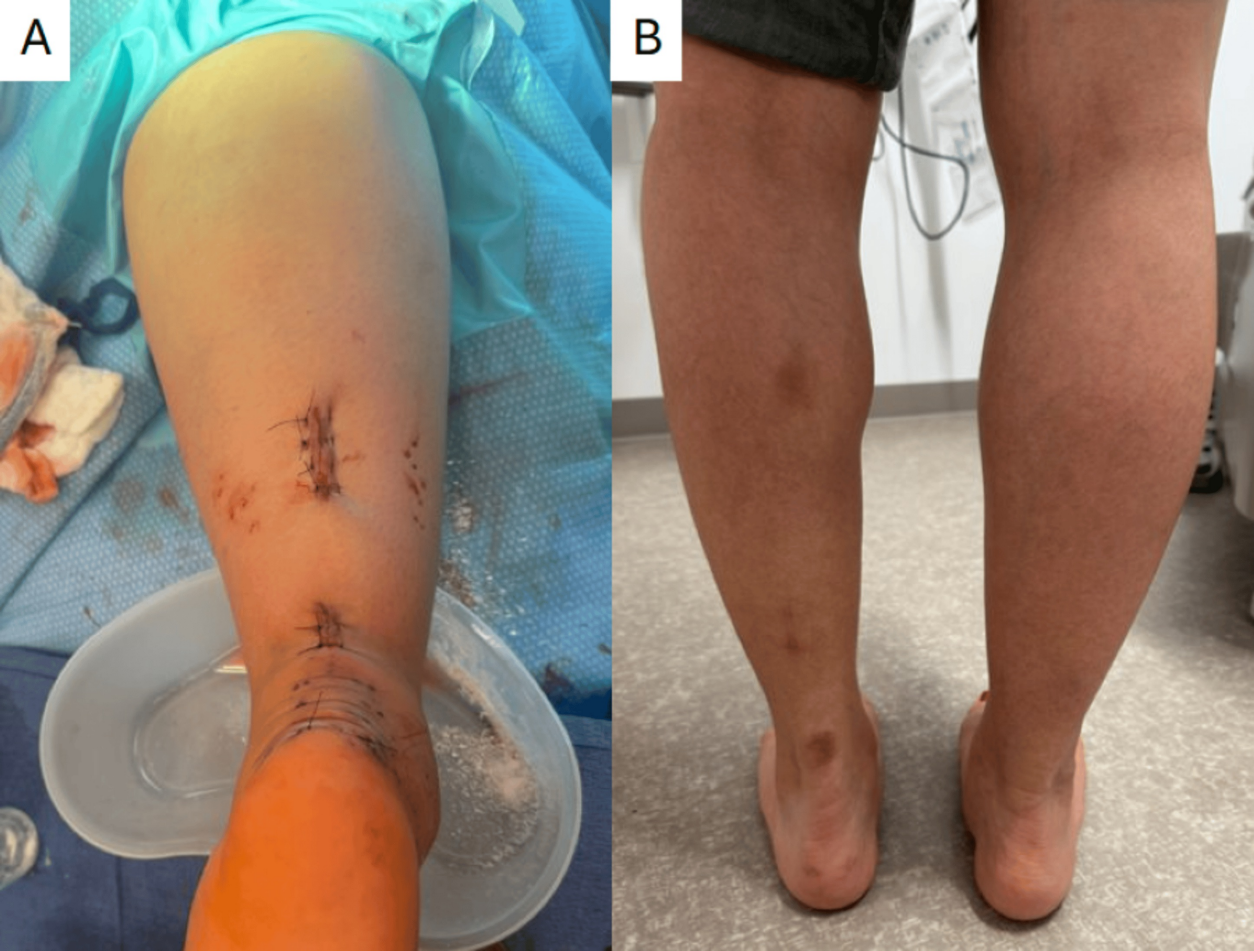
Intraoperative closure (A) and healed incisions one-year postoperatively (B).

The remainder of the case can be completed using a PARS-to-PARS (suture-to-suture) repair or a PARS-SpeedBridge (suture to calcaneal suture anchor; Arthrex Inc., Naples, FL, USA) repair, using the four-strand repair suture limbs in place of the ones passed with the PARS jig. It is our preference to use a PARS-SpeedBridge approach, especially in cases of poor tendon quality, in order to obtain direct tendon-to-bone fixation, bypassing suture knots and bulk in the area of the primary rupture. Anchors are drilled and tapped along the insertion of the Achilles tendon under fluoroscopic guidance before passing the suture through the distal stump and seating the anchors with the ankle in maximal plantarflexion (Figure 3). The paratenon is carefully closed in both the proximal and distal incisions to assist with vascular restoration and tendon healing. The postoperative course is the same as that of other patients at our institution undergoing PARS-SpeedBridge Achilles tendon repair (Table 1), with no additional restrictions. An example of the intraoperative closure and subsequent healed incisions for our technique is shown in Figure 4.

**Table 1:** Postoperative protocol for Achilles mid-substance PARS-Speedbridge repair. CAM, Controlled Ankle Motion; PARS, Percutaneous Achilles Repair System

Postoperative Time	Activity Restriction
Weeks 0-2	Non-weight-bearing in plantarflexion splint
Weeks 2-4	Non-weightbearing in CAM boot with heel lifts, dorsiflexion to neutral
Weeks 4-6	Progressive weightbearing and removal of lifts in the CAM boot, isometric calf exercises
Weeks 6-8	Weightbearing as tolerated in CAM boot, no dorsiflexion past neutral, graduated resistance exercises
Weeks 8-12	Wean into regular shoes, bilateral calf raises while seated, passive stretching, and continue with no dorsiflexion past neutral
Weeks 12-16	Plyometric training, single-leg raises, jogging, full range of motion without restriction
Months 6-8	Consider full running, agility, jumping with progressive return to sport

## Discussion

This mini-open conversion technique is ideal for salvaging cases of Achilles tendon rupture in which the PARS jig fails due to suture pull-out through poor-quality tendon. Many patients have pre-existing Achilles tendinosis, resulting in more fibrotic and scarred tissue that is not as robust and may lead to more shredding upon injury. One study found that up to 77% of patients showed evidence of preceding Achilles tendinosis at the rupture site [6].

Other contributors to suture pull-out include more complex tear morphology. In one study’s cohort, most mid-substance ruptures were found to be transverse; however, 10% of cases were found to have a more involved pattern, including coronal splits resulting in double-layers or Z-shaped ruptures [7]. The PARS jig is difficult to apply to tendons with these non-transverse rupture patterns, as the prongs may slip through the multiple layers, and passing needles may push aside rather than pierce the tissue.

The core weave stitch reduces the bulk of suture material on the tendon surface and has no significant difference in load to failure when compared with a Krackow stitch [8]. We have had success with both stitch techniques. 

## Conclusions

Overall, minimally invasive Achilles repair techniques such as PARS-to-PARS and PARS-Speedbridge offer several advantages, such as decreased wound complications and faster return to baseline activities compared with traditional open techniques. However, not all Achilles ruptures are amenable to the use of the PARS technique. The conversion technique described here has been performed in multiple cases where previously unrealized poor-quality tendon has been encountered, preventing the need for conversion to an extensile open repair.
